# Microbiota Transplantation as an Adjunct to Standard Periodontal Treatment in Periodontal Disease: A Systematic Review

**DOI:** 10.3390/medicina60040672

**Published:** 2024-04-21

**Authors:** Cherry Erlin Lindo, James Sebastian, Karina Natalie Kuntjoro, Valencia Audrey Halim, Fatimah Maria Tadjoedin, Sandra Olivia Kuswandani, Benso Sulijaya

**Affiliations:** 1 Periodontology Specialist Program, Department of Periodontology, Faculty of Dentistry, Universitas Indonesia, Jakarta Pusat 10430, Indonesia; cherry.erlin@ui.ac.id (C.E.L.); james.sebastian@ui.ac.id (J.S.); karina.natalie@ui.ac.id (K.N.K.); valencia.audrey31@ui.ac.id (V.A.H.); 2 Department of Periodontology, Faculty of Dentistry, Universitas Indonesia, Salemba Raya No. 4, Jakarta Pusat 10430, Indonesia; fatimah.tadjoedin@ui.ac.id (F.M.T.); sandra.olivia01@ui.ac.id (S.O.K.); 3 Graduate Research Program, UCL Eastman Dental Institute, Gower St., London WC1E 6AE, UK; 4 Dental Division, Universitas Indonesia Hospital, Depok 16424, West Java, Indonesia

**Keywords:** periodontitis, periodontal disease, microbiota transplantation

## Abstract

Periodontitis is a disease linked to severe dysbiosis of the subgingival microbiome. The treatment of periodontitis aims to change the dysbiosis environment to a symbiosis environment. We hypothesized that oral microbiota transplantation can lead to a significant improvement in periodontitis. Therefore, the aim of this study was to determine the effectiveness of microbiota transplantation after standard periodontal treatment in periodontitis patients. The search strategy was carried out by using the Boolean term “AND” to combine the keywords, which were “periodontitis AND microbiota transplantation”. Due to the limited resources of the study, we included both in vitro and in vivo investigations in this systematic review. The QUIN risk of bias tool was employed to assess the risk of bias in in vitro studies, while SYRCLE’s risk of bias assessment was used for in vivo studies. Oral microbiota transplants (OMTs) have shown potential in treating periodontitis. OMTs significantly reduced periodontitis-associated pathogenic microbial species (*P. endodontalis, Prevotella intermedia*, *T. vincentii*, *Porphyromonas* sp.) and increased beneficial bacteria (*P. melaninogenica, Fusobacterium nucleatum*, *P. catoniae, Capnocytophaga ochracea*, *C. sputigena*, *C. gingivalis*, *Haemophilus parainfluenzae*, and *Neisseria elongata)* upon in vitro testing. Furthermore, in the in vivo tests, single adjunctive OMT also had an effect on the oral microbiota composition compared to the full-mouth mechanical and antimicrobial debridement. OMTs may be cheaper and more effective at addressing high-risk individuals. At present, it is not possible to provide OMT clinical advice due to the lack of available information. This treatment needs to be subjected to more safety and efficacy testing before being included human clinical trials.

## 1. Introduction

Humans and bacteria have evolved together in symbiotic interactions over thousands of years [1]. Hundreds of different microbial species, including bacteria, fungus, viruses, and archaea, cohabit in the oral microbiome in well-organized ways [2]. There are both symbiosis and dysbiosis microorganisms. A symbiotic microbial benefit the host, but excessive pathogen growth can disrupt its balance, increasing the harmfulness of the adjacent microbial population and creating dysbiosis [3]. This, in turn, worsens the immunological responses of the host and leads to inflammatory consequences, which has the potential to induce chronic illnesses such as periodontal diseases [1,4].

Periodontal diseases are chronic inflammatory conditions that affect the tissues surrounding and supporting the teeth. Their occurrence is predicted to affect approximately 45–50% of the global population, with 11.2% of individuals being diagnosed with severe periodontitis. Periodontal diseases typically arise from the presence of dental plaque, which is a microbial biofilm. These diseases are linked to the proliferation of various bacterial species which occur as a result of the dysbiosis of the plaque [5,6]. Numerous studies have explored *Porphyromonas gingivalis*, *Tannerella forsythia, Treponema denticola*, and *Aggregatibacter actinomycetemcomitans’* pathogenicity for periodontitis [6]. Evidence from animal models suggested that these bacteria could alter the host’s signaling pathways, leading to the disruption of tissue homeostasis. A compromised innate immune system might result in alterations in the prevalence of bacteria, which promotes inflammation and bone degradation [7].

Conventional periodontal therapy causes alterations in the microbial ecology, but these changes are temporary [8,9]. Many studies have stated that having a suitable microbial ecosystem is crucial for preserving resistance to infection. Recently, there has been growing interest in using a new approach called fecal microbiota transplantation (FMT) to modify imbalanced gut bacteria in the treatment of intestinal diseases [10]. In addition to intestinal diseases, other conditions that may include a disruption in microorganism balance, such as inflammatory bowel disease and obesity, have demonstrated favorable outcomes with microbial transplantation [11].

Inspired by the success of FMT, this study introduces a form of microbial transplantation known as oral microbiota transplantation (OMT) as a potential therapy for periodontitis, which is also an inflammatory disease. OMT is similar in concept to a fecal microbiome transplant, which involves transferring oral bacteria from a healthy donor to a patient suffering from a disease such as periodontitis. Despite the known association between periodontitis and the significant disruption of the subgingival microbiome, there is currently no scientific evidence or systematic reviews supporting the use of OMT as a treatment for periodontal disease in humans. We hypothesized that OMT improves periodontal clinical measures more than conventional periodontal treatment in periodontitis. Therefore, this systematic review aimed to assess the safety and effectiveness of OMT as an adjunctive treatment to standard periodontal treatment.

## 2. Method

This systematic review followed the guidelines of the Preferred Reporting Items for Systematic Reviews and Meta-Analysis (PRISMA). The review was conducted by searching the studies that have been published in Wiley, Science Direct, Scopus, EBSCO, and PubMed. The subjects of this study were studies that included comparisons of the effectiveness of oral microbiota transplantation compared to standard periodontal treatment in periodontitis patients in all species. The inclusion criteria were all studies addressing microbiota transplantation, including studies in vitro studies, in vivo studies, clinical studies, and case reports, after standard periodontal treatment in periodontitis patients in all species; publications from the last 10 years; full papers published in English; studies with results regarding periodontal pocket depth, bleeding on probing, and/or plaque index; and studies with participants with a probing depth of at least 5 mm for periodontitis patients. We used the last 10 years as the publication filter because OMT is new and understudied; we tried expanding the search period to 20 years but discovered nothing related to our topic [12]. The exclusion criteria were studies in the form of systematic reviews, meta-analyses, and case series; studies with participants with systemic disease; and studies with smoking participants. Because they are forms of secondary research, systematic reviews and meta-analyses were excluded [13]. Given the absence of a control group and the risk of bias, case series were considered a weaker form of evidence and were therefore excluded [14]. We also excluded participants with systemic disease and smokers because these are known periodontitis risk factors that can affect treatment success and prognosis [15].

The clinical question for this review was “How is the potential of Oral Microbiota Transplant (OMT) as an adjunctive treatment for periodontitis?”. This clinical question was translated using PICO (Population, Intervention, Comparison, and Outcome). A detailed description of this study’s PICO can be seen in Table 1. The keywords were arranged using a combination of the words periodontitis and microbiota transplantation. The search strategy was carried out by using the Boolean term “AND” to combine the keywords “periodontitis AND microbiota transplantation”. This review protocol is recorded in the PROSPERO database under the number 492,313 for the systematic review of animal clinical trials and CRD42023492327 for the systematic review of human clinical trials. The keywords consisted of “systematic review”, “periodontitis”, and “microbiota transplantation”.

The study selection process was carried out manually using Excel ver. 2403. Duplicate search results from different databases were excluded. Furthermore, inappropriate titles and abstracts were excluded. The studies obtained were reviewed to assess whether they met the specified inclusion and exclusion criteria and were then assessed for the risk of bias using the QUIN tool ver. 1.0 and SYRCLE’s ROB Tool ver. 1.0. Four people carried out the entire systematic review procedure, which included the literature search; abstract, title, and full-text screening; and qualitative data extraction. Discussions were held by the reviewers to resolve disagreements in the selection of the studies. If consensus was not achieved, well-experienced supervisors were consulted to make the final decision.

## 3. Result

### 3.1. Research Identification and Selection

**Figure 1:**
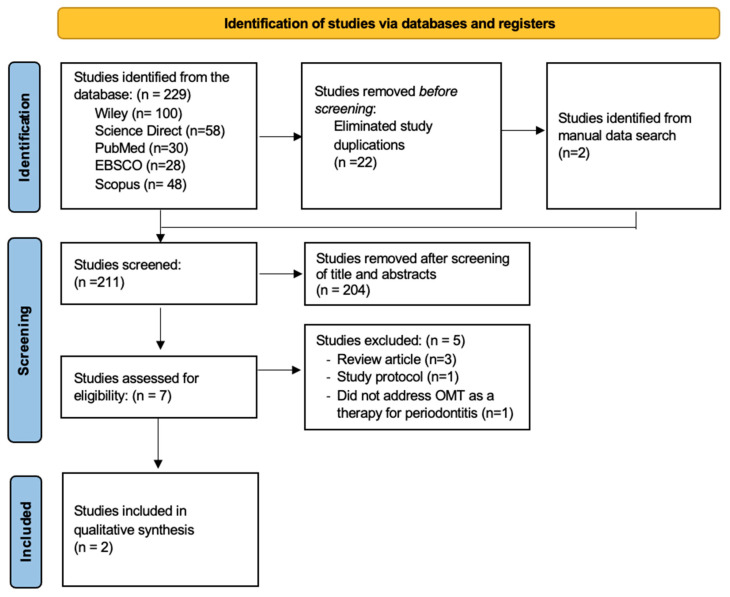
PRISMA flow diagram.

The research selection process adhered to the PRISMA (Preferred Reporting Items for Systematic Reviews) recommendations. A flow chart is provided in Figure 1. The process of research identification began with a thorough search on five electronic databases, including Wiley, ScienceDirect, PubMed, Scopus, and EBSCO. This search was conducted using a combination of keywords, including microbiota transplantation and periodontitis.

The search conducted on five electronic databases using the specified keywords resulted in the identification of a total of 229 papers. More precisely, 100 studies were obtained from Wiley, 58 studies from ScienceDirect, 30 studies from PubMed, 28 studies from EBSCO, and 13 studies from Scopus. Two studies were obtained from alternative sources. All these studies were deduplicated manually using Excel, and a total of 22 repeated studies were identified. A total of 209 studies passed title and abstract screening, out of which 202 studies were discarded due to non-compliance with the specified inclusion and exclusion criteria established by the authors. Consequently, only seven studies remained, the whole texts of which were thoroughly examined.

After carrying out a comprehensive analysis of the entirety of each manuscript, three studies were excluded since they were review articles; one study was excluded as it was a study protocol; and one study did not address the topic of OMT for therapy in periodontitis. A total of two studies were included for qualitative synthesis.

### 3.2. Risk of Bias Assessment

The risk of bias assessment for the in vitro studies was conducted using the QUIN risk of bias tool ver. 1.0, which has twelve domains. The assessment results were classified as low risk of bias, some concern, and high risk of bias [16]. Meanwhile, the animal studies (in vivo) were evaluated using SYRCLE’s RoB tool ver. 1.0, which is derived from the Cochrane RoB tool ver. 2.0 and has been modified to address biases that are particularly relevant in animal intervention studies. The RoB tool developed by SYRCLE comprises eleven dimensions, with the evaluation outcomes classified as low risk of bias, high risk of bias, or uncertain risk of bias [17].

**Figure 2:**
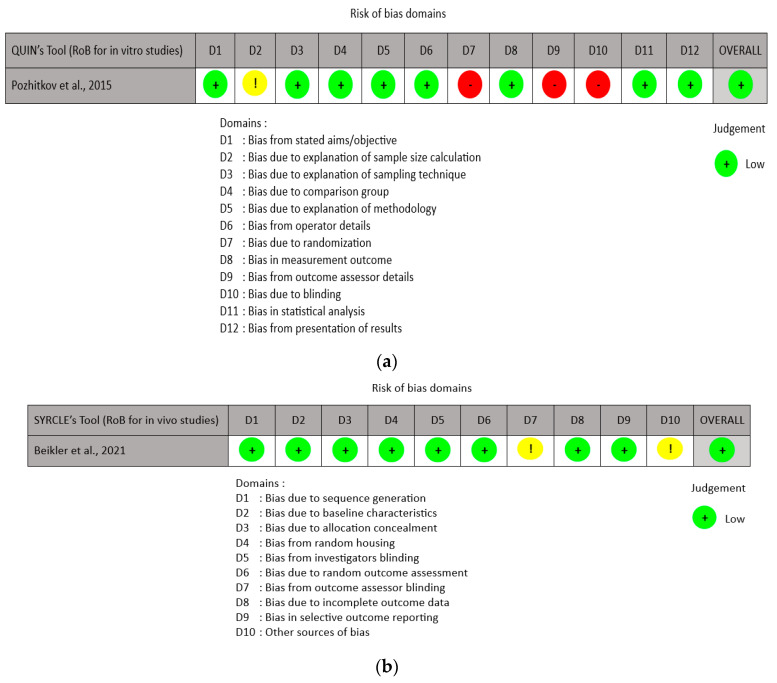
The results of the risk of bias assessments: (**a**) Assessment of the risk of bias for in vitro studies [18] using the QUIN RoB tool. (**b**) Assessment of the Risk of Bias for in vivo studies [1] using SYRCLE’s RoB tool.

The QUIN tool-based risk of bias evaluation revealed that the in vitro studies have a low risk of bias. According to the SYRCLE’s risk of bias tool-based assessment, the in vivo studies were also deemed to have a low risk of bias. Figure 2 presents the outcomes of the risk of bias evaluations.

### 3.3. Qualitative Synthesis

Qualitative synthesis was carried out on two studies by extracting important data from each study. The author extracted some data, namely, author’s name, year of publication, subject and sample size, intervention, periodontal clinical parameters, duration of follow-up, and results, which are summarized in Table 2.

## 4. Discussion

Complex populations of bacteria known as microbiota are found and colonize on our mucosal surfaces. These complexes are specifically tailored to the various environmental niches found in the human body, including the mouth. The human microbiome, which is made up of the microbiota on our mucosal surfaces and other anatomical locations in the body, has been the subject of extensive research in recent years. This is due to the realization that the homeostasis between these organisms and the human host is fundamental to our biology, the preservation of our health, and the onset of disease. This homeostasis condition refers to ‘symbiosis’, while a detrimental change to a microbiome that is no longer in balance with the host is known as ‘dysbiosis’ [19].

The second largest and most studied microbiome is the oral microbiome. Homeostasis must be preserved to maintain a healthy oral ecosystem; however, changes such as puberty, bad dental hygiene, alcohol and tobacco use, hormonal imbalances, and stress can break this homeostasis and cause a variety of illnesses, including oral diseases such as periodontal disorders [20]. The pathogenesis of periodontal disorders, including the host response, and the oral microbiome interact in a complicated way. Periodontal disorders, including gingivitis and periodontitis, are persistent inflammatory conditions that impact the tissues that support teeth. Globally, their prevalence is believed to be between 45 and 50% of people, with severe periodontitis accounting for 11.2% of cases [6].

**Figure 3:**
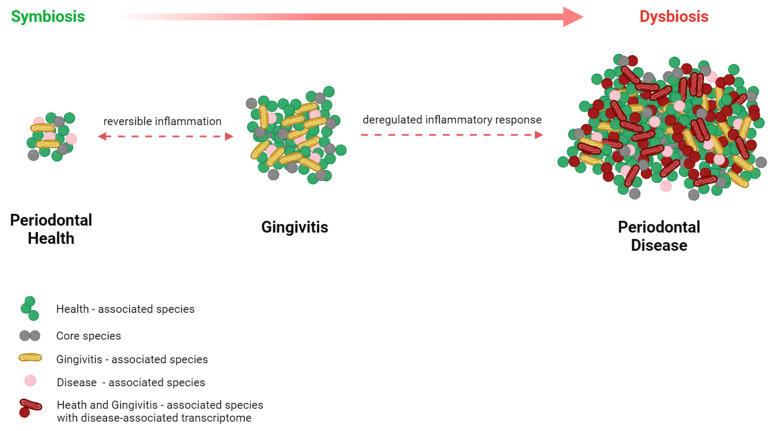
The bidirectional relationship between the subgingival microbiome and the inflammatory and immune response.

The European Federation of Periodontology and the American Academy of Periodontology both describe gingivitis as a common oral infection that is characterized by swelling, redness, and inflammation of the soft tissues around the teeth caused by dental plaque [21]. Prolonged inflammation can promote the progression of periodontal pockets, altering the nutritional conditions and enhancing the variety and abundance of biofilms, leading to dysbiosis [22]. Failure to undergo gingivitis treatment may lead to the progression of periodontitis, an inflammatory disease characterized by the formation of periodontal pockets, the loss of alveolar bone, and tooth loss [23,24]. The bidirectional relationship between the subgingival microbiome, which contributes to the development of gingivitis and periodontal disease, is illustrated in Figure 3.

Eliminating biofilm and dental plaque has been suggested to reduce the advancement of periodontitis and gingivitis as they are the primary contributors to these conditions. Treatments for periodontal disease include the use of antimicrobials, root planing, scaling, and deep pocket debridement, all of which are aimed at diminishing the presence of harmful microorganisms [19,25,26,27]. Scaling and root planing are the standard non-invasive therapies for periodontitis that effectively reduces microbiological levels and bleeding levels after probing by eliminating the subgingival biofilm, calculus, and bacterial toxins present on the cementum surface [19,25,26,27]. Unfortunately, due to the bacteria’s ability to recolonize during an 8-week therapy period, scaling and root planing are not adequate in many instances. Consequently, it has been recommended to apply an adjunct of treatment techniques alongside scaling and root planing, including systemic/local antimicrobials, antimicrobial Photodynamic Therapy (aPDT), or host modulation therapy [27].

Recently, researchers have been exploring a promising novel treatment for periodontal disease, oral microbiota transplantation (OMT). This treatment employs a similar approach to a fecal microbiome transplant (FMT), which involves the transfer of oral microbiota from a healthy person to a periodontal disease patient. The objective is to encourage the growth of good bacteria that can aid in the control of inflammation, stop more damage occurring to the periodontal tissues, and restore the healthy homeostasis of the oral microbiota, which is disturbed in periodontal disease [28,29].

Recently, OMT therapy has been effectively administered to dogs that have naturally developed periodontitis [1]. However, this process has not yet been finalized in human subjects. Logistical factors must be considered when carrying out direct microbiome transplantation in humans, which involves transferring microorganisms from a donor to a recipient [4,5]. A study by Nath S. et al. (2021) proposed a novel approach for developing and testing OMT prior to human clinical trials. The overall strategy includes (1) identifying healthy donor plaque material and developing the plaque microbiota in vitro; (2) developing a delivery system for OMT therapy; and (3) transplanting donor material into an animal model to verify its efficacy and safety [4].

**Figure 4:**
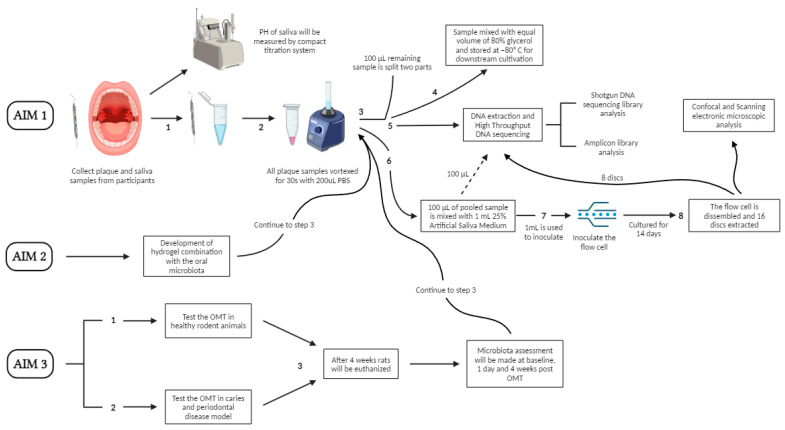
OMT flow diagram.

Firstly, it is necessary to identify the oral microbiome plaque in healthy donors. Then, it is necessary to select participants who are both systemically and periodontally healthy, with probing depth less than 4 mm, no clinical attachment loss, and less than 20% bleeding on probing (BOP). Participants’ plaque and saliva samples are obtained during these procedures. For dental plaque collection, samples are taken using a dental curette from four sites (the buccal and lingual surfaces of the upper and lower central incisors and the mesio-buccal surfaces of the molars). The samples of plaque are mixed with 200 μL of phosphate-buffered solution. For saliva collection, participants spit into a graded saliva collection tube. The samples from the first two minutes are discarded; the ones from the next five are preserved for examination. The samples are subsequently transferred to an Oral Microbiology Laboratory for in vitro biofilm growth and inoculation. The second step is the development of an OMT therapeutic delivery system. In one study protocol, the oral microbiota were combined with a hydrogel. The final stage involves assessing the microbiota and testing the OMT in animals with and without periodontitis [4]. A flow diagram of the OMT protocol is displayed in Figure 4.

According to Pozhitkov, AE. et al., OMT may be a new alternative for treating periodontitis. Their study used 16 systemically healthy white adults with various oral conditions, such as periodontitis, caries, edentulism, and other oral health conditions. The oral microbiota in individuals with periodontitis exhibited much higher diversity compared to those observed in healthy individuals or those with caries and edentulous conditions. While a complex of *P. melaninogenica*, *Fusobacterium nucleatum*, *P. catoniae*, *Capnocytophaga ochracea, C. sputigena*, *C. gingivalis*, *Haemophilus parainfluenzae*, and *Neisseria elongata* contributed to the microbial composition of oral healthy subjects, *P. endodontalis*, *Prevotella intermedia*, *T. vincentii*, and an uncultured *Porphyromonas* sp. were found to contribute to the microbial composition in periodontitis [18]. This finding offers an opportunity for the development of an innovative therapy for periodontitis involving the replacement of the disease-related microbiome with a microbiome associated with good health [5,18].

In a study by Beikler et al., eighteen beagle dogs with a natural history of periodontitis but in good health were randomized to the test or control group. The universal OMT donor was a 4-year-old female beagle dog with good periodontal health. Oral microbiota transplants from the healthy donor were given to the test dogs. Clinical exams and whole-mouth oral microbiota samples were taken at week 2, baseline, week 2, and week 12. High-throughput 16S ribosomal RNA gene amplicon sequencing, taxonomic assignment, and bioinformatic and statistical analyses were used to investigate the oral microbiota samples. The key outcome measure, pocket depth at week 12, showed no significant difference with the control group, but single adjunctive OMT affected oral microbiota composition in a better way than full-mouth mechanical and antibacterial debridement. No local or systemic adverse effects occurred throughout the study period [1].

According to the study of Utter et al. [30], when it comes to highly resistant oral microbial populations, a single transplant might not be enough to bring about long-lasting changes. To maintain beneficial alterations to the recipient’s imbalanced oral microbiota and improve clinical outcomes, it may be necessary to administer OMT multiple times [1,30]. The safety issues associated with the possible use of OMTs are comparable to those for oral probiotics. Similar to probiotics, transplanted biofilms must not induce disease and should exhibit a high level of genetic stability [5]. Based on recent studies, we can determine that OMT can be beneficial as an adjunct to periodontal therapy. OMT could be utilized for patients in the early stages of their disease to prevent the disease’s progression by promoting the formation of a healthy microbiome [18]. The limitations of this study include the fact that only two studies have been completed on the application of OMT in periodontitis, and neither of them evaluated OMT in human patients. Due to the limited scope of this study, it is challenging to make definitive conclusions. Further research into OMT for periodontal disease is required.

## 5. Conclusions

Despite the small number of studies in the literature on OMT, this study contributes valuable insights into the potential benefits of OMT in treating periodontitis. OMT has the potential to be more cost-effective and efficient in targeting people with a higher risk of disease. This study contributes to the investigation of this new therapeutic approach for treating periodontitis. However, due to the aforementioned limitations, it is not possible to provide OMT-related clinical advice due to the lack of available information. Future research should prioritize addressing these information gaps to further our understanding of OMTs and their significance in periodontitis treatment. Additional safety and efficacy examinations will be needed before this therapy can be tested in human clinical trials.

## Figures and Tables

**Table 1:** PICO description.

Population (P)	Intervention (I)	Comparison (C)	Outcome (O)
Periodontitis in all species	Standard periodontal treatment with oral microbiome transplantation	Standard periodontal treatment without oral microbiome transplantation	Primary outcome: Microbial composition Secondary outcome - Probing pocket depth (PPD) - Bleeding on probing (BOP) - Plaque index (PI)

**Table 2:** Qualitative synthesis results.

Author (Year)	Title	Subject	Study Design	Intervention	Periodontal Clinical Parameter	Follow-up/Evaluation	Result
Beikler et al., 2021 [1]	Oral Microbiota Transplant in Dogs with Naturally Occuring Periodontitis	18 male and female dogs, ages 2–7 years old.	Randomized controlled trial(In vivo)	Full-mouth SRP + glycine powder air polishing + subgingival and oral irrigation with 0.1% NaOCl, followed by OMT in the test group.	- Primary: microbiota composition - Secondary: probing pocket depth (PPD), bleeding on probing (BOP), plaque index—O’Leary (PI)	- After OMT - 2 weeks after OMT - 12 weeks after OMT	- Clinical parameters: no significant intergroup differences at any time point. - Microbiota composition: single adjunctive OMT had an additional effect on the oral microbiota composition compared to the full-mouth mechanical and antimicrobial debridement alone.
Pozhitkov et al., 2015 [18]	Towards microbiome transplant as a therapy for periodontitis: an exploratory study of periodontitis microbial signature contrasted by oral health, caries and edentulism	16 systemically healthy male and female white adults aged 28–58 years old.	Exploratory and experimental (in vitro)	- Collecting oral biofilm samples. - Volunteers’ oral plaque was plated on blood agar after saline, 16 mM NaOCl, and ascorbate buffer neutralization. - DNA and165 rRNA genes were amplified.	Microbial composition		- Statistically significant microbial signatures of orally healthy subjects compared to periodontitis. - This finding presents a path forward for a potential new therapy for periodontitis which could be based on substituting periodontitis-associated microbiomes with the health-associated ones.
